# The Epidemiology of Urinary Tract Trauma: Results from the GRAND Study

**DOI:** 10.3390/jcm14155343

**Published:** 2025-07-29

**Authors:** Nikolaos Pyrgidis, Julian Marcon, Gerald Bastian Schulz, Patrick Keller, Yannic Volz, Lennert Eismann, Robert Bischoff, Paulo L. Pfitzinger, Michael Chaloupka, Christian Stief, Philipp Weinhold

**Affiliations:** Department of Urology, University Hospital of the LMU Munich, 81377 Munich, Germany; julian.marcon@med.uni-muenchen.de (J.M.); gerald.schulz@med.uni-muenchen.de (G.B.S.); patrick.keller@med.uni-muenchen.de (P.K.); yannic.volz@med.uni-muenchen.de (Y.V.); lennert.eismann@med.uni-muenchen.de (L.E.); robert.bischoff@med.uni-muenchen.de (R.B.); paulo.pfitzinger@med.uni-muenchen.de (P.L.P.); michael.chaloupka@med.uni-muenchen.de (M.C.); christian.stief@med.uni-muenchen.de (C.S.); philipp.weinhold@med.uni-muenchen.de (P.W.)

**Keywords:** kidney trauma, ureteral trauma, bladder trauma, urethral trauma, epidemiology, perioperative outcomes

## Abstract

**Background:** Urinary tract trauma encompasses injuries to the kidneys, ureters, urinary bladder, and urethra and can result from both external and iatrogenic causes. We aimed to evaluate the epidemiology, clinical characteristics, and in-hospital outcomes of urinary tract trauma in Germany. **Methods:** We analyzed data from the GeRmAn Nationwide inpatient Data (GRAND) registry, provided by the Research Data Center of the Federal Bureau of Statistics, from 2005 to 2023. We included patients admitted to the hospital with kidney, ureteral, urinary bladder, or urethral trauma. We assessed baseline characteristics, perioperative outcomes, surgical interventions, in-hospital all-cause mortality, and trends. **Results:** We identified 239,657 patients with urinary tract trauma: 109,376 with kidney, 34,330 with ureteral, 57,886 with bladder, and 38,065 with urethral trauma. While the incidence of kidney trauma declined, the incidence of ureteral, bladder, and urethral trauma steadily increased over time. Kidney trauma was the most common trauma, affecting younger males (median age of 47 years), and was associated with in-hospital all-cause mortality of 2.4% and transfusion rates of 15%. Ureteral stenting was necessary in 9.3% and nephrectomy in 2.6% of all patients with kidney trauma. Moreover, ureteral, bladder, and urethral trauma predominantly affected older, multimorbid patients, leading to higher rates of transfusion (22–25%), intensive care unit admission (12–15%), and mortality (3.2–6.4%). Ureteral anastomosis was necessary in 14% of all ureteral injuries. Bladder repair was required in 53% of all patients with bladder injury, while 1% of these patients required cystectomy. Accordingly, urethral reconstruction was performed in 7.2% of all patients with urethral trauma. **Conclusions:** These findings highlight the evolving landscape of urinary tract trauma and underscore the need for tailored management strategies and preventive measures.

## 1. Introduction

Urinary tract trauma encompasses a diverse spectrum of injuries that may involve the kidneys, ureters, urinary bladder, or urethra and can result from both intentional (interpersonal violence, self-inflicted injury) and unintentional causes (road traffic or other domestic accident, fall) [1]. Its etiology ranges from high-energy blunt and penetrating injuries in younger individuals to an increasing proportion of iatrogenic and fragility-related injuries in elderly patients undergoing diagnostic or therapeutic urological or non-urological abdominal procedures [2].

Renal trauma is the most frequent genitourinary trauma and is present in up to 5% of all cases [3]. Most renal injuries can be managed non-operatively with successful organ preservation [4]. On the contrary, ureter trauma is relatively rare as the ureters are protected by their small diameter, mobility, and posterior location, as well as by the adjacent musculoskeletal and visceral structures [5]. Iatrogenic injury during surgery is the most common cause of ureteral injury and may result in significant morbidity when left intraoperatively untreated [6]. Urinary bladder trauma is typically caused by pelvic crush or blow to the lower abdomen and is associated, in most cases, with pelvic fractures and other intra-abdominal injuries [7]. Therefore, urinary bladder trauma is associated with significant morbidity, prolonged hospitalization, and complex surgical management [8]. Urethral trauma is also associated with concomitant pelvic or penile fractures [9]. If not properly treated, urethral trauma can lead to significant short- and long-term complications [10].

Despite the clinical relevance of urinary tract trauma, current epidemiological data remain limited, particularly at the national level. Most available studies originate from single institutions or trauma registries, often focusing on a specific anatomical site or trauma mechanism [11]. A contemporary, population-based assessment is mandatory to identify at-risk patient populations, capture the shifting patterns in urinary tract trauma, and highlight the emerging clinical challenges associated with its preventative strategies and management guidelines [12]. In this context, we conducted a comprehensive, large-scale analysis of the GeRmAn Nationwide inpatient Data (GRAND), aiming to evaluate the epidemiology, clinical characteristics, and in-hospital outcomes of patients admitted with kidney, ureteral, bladder, or urethral trauma in Germany in recent years.

## 2. Methods

### 2.1. GRAND Registry

This analysis was based on nationwide inpatient data collected from all German hospitals between 2005 and 2023. The dataset includes anonymized information on patient comorbidities, surgical interventions, and perioperative outcomes, covering all hospitals in Germany, except for psychiatric, forensic, and military facilities. These data were compiled and managed by the German Federal Statistical Office (Wiesbaden, Germany) and were accessed under an approved data use agreement (LMU—4710-2022). Since the introduction of the German Diagnosis-Related Group (G-DRG) system in 2004, hospitals have been required to report inpatient treatment data to the Institute for the Hospital Remuneration System for billing purposes. Comorbidities and complications were coded according to the International Classification of Diseases, 10th Revision, German Modification (ICD-10-GM), while surgical procedures followed the German Procedure Classification (OPS). To ensure consistency in coding, the German Institute for Medical Documentation and Information provides standardized coding guidelines.

### 2.2. Study Population and Outcomes

This study included patients who were admitted to the hospital with kidney trauma (ICD-10 code: S37.0), ureteral trauma (ICD-10 code: S37.1), urinary bladder trauma (ICD-10 code: S37.2), or urethral trauma (ICD-10 code: S37.3). To capture baseline characteristics, perioperative complications, and surgeries, additional ICD-10 and OPS codes were utilized. The primary outcome of the present study was to evaluate the epidemiology of urinary tract trauma. Secondary outcomes included the baseline characteristics, perioperative complications, and surgeries, as well as the admissions to the intensive care unit (ICU) of these patients in Germany.

### 2.3. Statistical Analysis

All statistical analyses were conducted on our behalf at the Research Data Center of the German Office of Statistics using R scripts developed by our research team (source: Research Data Center of the German Office of Statistics, DRG Statistics 2005–2023, R statistical software Version 4.1.2, own calculations). Since only aggregated results were accessible to our research team and individual patient data remained confidential, ethical approval and patient consent were not required for the present study, following the German regulations. Categorical variables were reported as absolute numbers and percentages, while continuous variables were presented as median values with interquartile range (IQR).

## 3. Results

### 3.1. Kidney Trauma

**Figure 1:**
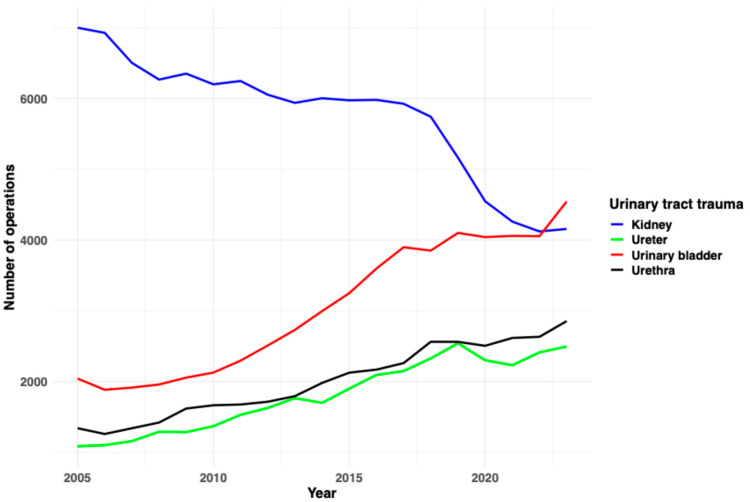
The annual trends of urinary tract trauma in Germany from 2005 to 2023.

A total of 109,376 patients were hospitalized due to kidney trauma primarily between 2005 and 2023. The majority of these patients were male (70%), with a relatively young median age of 47 years (IQR: 24–65). Kidney trauma was predominantly observed in younger age groups, with 31% of cases occurring in individuals under 30 years old. The overall median length of hospital stay was 4 days (IQR: 2–10). Relevant comorbidities included hypertension in 23%, diabetes in 8.1%, and chronic kidney disease in 7.1% of cases. Acute kidney failure was documented in 5% of all patients, blood transfusions were required in 15% of cases, and 13% of all patients were admitted to the ICU (Table 1). A total of 10,209 (9.3%) patients required ureteral stenting, 2812 (2.6%) required nephrectomy, and 1989 (1.8%) required kidney embolization. In-hospital all-cause mortality was 2.4%. The incidence of kidney trauma has declined over the years, with 7001 cases hospitalized in 2005 compared to 4156 in 2023 (Figure 1). The incidence per 100,000 German inhabitants of kidney trauma is presented in Table 2.

### 3.2. Ureteral Trauma

In total, 34,330 patients were admitted with ureteral trauma, displaying a median age of 60 years (IQR: 48–72). Only 42% of these patients were male. Patients with ureteral trauma had a high burden of comorbidities, including hypertension (37%), diabetes (12%), and chronic kidney disease (9.3%). Their median length of hospitalization was 9 days (IQR: 3–18). Acute kidney failure was documented in 8.9% of all patients, blood transfusions were required in 22% of cases, and 13% of all patients were admitted to the ICU (Table 1). A total of 16,421 (48%) patients required ureteral stenting, 4846 (14%) required ureteral anastomosis, and 510 (1.5%) required nephrectomy. Accordingly, the overall in-hospital all-cause mortality rate was 3.2%. The incidence of ureteral trauma has increased over the years, with 1085 cases hospitalized in 2005 compared to 2493 in 2023 (Figure 1). The incidence per 100,000 German inhabitants of ureteral trauma is presented in Table 2.

### 3.3. Urinary Bladder Trauma

A total of 57,886 patients were hospitalized due to urinary bladder trauma. The median patient age was 63 years (IQR: 47–76), and only 40% were male. Comorbidities were common, with hypertension observed in 37%, diabetes in 12%, chronic kidney disease in 9.6%, and chronic heart failure in 7.2%. The median length of stay was 10 days (IQR: 6–17). Acute kidney failure occurred in 9.1%, while 25% of patients required transfusions, and 15% of cases were admitted to the ICU (Table 1). Accordingly, the in-hospital all-cause mortality rate was 5.2%. Surgical repair was common, with 30,604 (53%) patients undergoing bladder suturing and 570 (1%) undergoing cystectomy. Patients undergoing cystectomy had higher mortality rates. The incidence of urinary bladder trauma has increased over the years, with 2041 cases hospitalized in 2005 compared to 4544 in 2023 (Figure 1). The incidence per 100,000 German inhabitants of urinary bladder trauma is presented in Table 2.

### 3.4. Urethral Trauma

Among 38,065 patients with urethral trauma, the majority (94%) were male. This group was characterized by an older patient profile, with a median age of 74 years (IQR: 58–83), and 39% of patients were over the age of 80. Comorbidities included hypertension (50%), diabetes (21%), chronic kidney disease (20%), and chronic heart failure (22%). The median hospital stay was 9 days (IQR: 4–18). Acute kidney failure occurred in 12%, while 18% of patients required blood transfusion, and 12% of cases were admitted to the ICU (Table 1). Urethral reconstruction was performed in 2756 (7.2%) patients. Interestingly, the in-hospital all-cause mortality rate reached 6.4%, the highest in this cohort. The incidence of urethral trauma has also increased over the years, with 1339 cases hospitalized in 2005 compared to 2853 in 2023 (Figure 1). The incidence per 100,000 German inhabitants of urethral trauma is presented in Table 2.

## 4. Discussion

The present nationwide analysis provides an overview of the trends, epidemiology, and in-hospital outcomes of urinary tract trauma in Germany over the past two decades. Kidney trauma is the most frequently observed entity in Germany, predominantly affecting younger male patients. Despite their younger age, some of these patients required invasive interventions, such as nephrectomy or kidney embolization, underscoring the potential severity of these injuries in some cases. Interestingly, a steady decline in the annual incidence of kidney trauma was observed. In contrast, ureteral, urinary bladder, and urethral injuries were more commonly seen in older patients with a significantly higher comorbidity burden, which resulted in prolonged hospital stay and substantially elevated complication and mortality rates. Interestingly, their annual incidence is steadily increasing, suggesting a shift in the trauma spectrum from high-energy external injuries in younger individuals toward iatrogenic and fragility-related injuries in multimorbid elderly populations.

The findings of the present study in terms of renal trauma are in line with previous relevant studies. Blunt abdominal trauma is the most common mechanism of renal injury [13]. The favorable outcomes of renal injuries in this cohort highlight that most cases are low-grade and amenable to conservative management [14]. However, 2.6% of these patients still required nephrectomy, and only 1.8% underwent kidney embolization, emphasizing the need for careful risk stratification and appropriate imaging. On the contrary, ureteral injuries were less frequent but were associated with significant perioperative morbidity [15]. Previous studies indicate that ureteral trauma is rare but clinically challenging, often resulting from iatrogenic injury during pelvic or abdominal surgery [16]. Our data, showing high rates of stenting and ureteral anastomosis, support this, suggesting that a plethora of injuries are not immediately recognized and may require delayed intervention. Therefore, it is mandatory to identify these injuries intraoperatively to prevent complications such as urinoma, fistula, or strictures [17]. The relatively high ICU admission and mortality rates also underscore the systemic impact of undetected or inadequately managed ureteral trauma. Thus, it is of paramount importance to increase awareness, mainly among non-urological surgeons, to improve the outcomes of the management of ureteral trauma [18].

Bladder injuries were also associated with high in-hospital morbidity and mortality [19]. Bladder trauma typically results from pelvic fractures or iatrogenic causes during gynecological or urological procedures [20]. The high rate of surgical repair and significant comorbidity burden observed in this cohort suggest that both mechanisms are highly relevant in the German inpatient setting. Therefore, our findings reinforce the importance of early recognition and prompt the repair of intraperitoneal ruptures, as well as conservative management for select extraperitoneal injuries [21]. Accordingly, urethral injuries, while relatively rare, demonstrated a unique demographic profile, affecting predominantly elderly male patients with an exceptionally high comorbidity burden. These findings align with the available evidence, suggesting that urethral injuries are often iatrogenic or catheter-related in older men [22]. The low rate of direct urethral reconstruction aligns with the current guideline recommendations, highlighting the importance of initial catheter diversion and delayed urethroplasty, in most cases, reserving immediate repair for complex or complete disruptions in stable patients [23].

Despite the high number of cases and the use of standardized national hospital data, our study is not devoid of some important limitations. As with all studies based on administrative datasets, the accuracy of our findings depends on the precision of ICD-10 and OPS coding, which may be subject to errors or misclassification. Additionally, important clinical variables such as the mechanism of trauma, injury severity scores, imaging findings, and functional outcomes were not available in the GRAND registry. Similarly, information on timing and intent of the urinary tract trauma (e.g., iatrogenic versus blunt), as well as long-term follow-up data, including reoperations, recovery, and quality of life, was lacking. Importantly, the cause of the iatrogenic trauma (ureteroscopy, shockwave lithotripsy, or other urological procedures, as well as hysterectomy or other pelvic surgery) could not be explored. Moreover, we were unable to differentiate between isolated and concomitant injuries, and we could not account for differences in hospital volume, trauma center designation, or surgical expertise, all of which may have affected outcomes. Of note, we performed a descriptive analysis of urinary tract trauma, and it was beyond the scope of the present study to compare outcomes among the different urinary tract traumas. Finally, as the dataset is limited to inpatient admissions in Germany, our findings may not be directly generalizable to other healthcare systems or outpatient settings.

## 5. Conclusions

In this large-scale, real-world study spanning nearly two decades, we provide a detailed snapshot of the epidemiological patterns and in-hospital outcomes of urinary tract trauma in Germany. Kidney trauma predominantly affects younger males, whereas ureteral, bladder, and urethral injuries are increasingly encountered in elderly, multimorbid patients and are, thus, linked to higher complication rates and worse outcomes. These findings reflect a paradigm shift in urinary tract trauma, likely driven by aging populations and increased iatrogenic injuries. Our results underscore the need for age- and comorbidity-tailored management strategies for risk reduction, such as improved intraoperative identification of ureteral structures or targeted training for high-risk procedures among non-urologists, and highlight the importance of preventative measures, particularly in high-risk patient groups. Future prospective studies are warranted to validate these observations and to further investigate long-term outcomes following urinary tract trauma.

## Figures and Tables

**Table 1:** Baseline characteristics of patients with urinary tract trauma. Variables are presented as median with interquartile range or frequencies with proportions. ICU: intensive care unit.

Characteristic	Kidney Trauma, n = 109,376	Ureteral Trauma, n = 34,330	Urinary Bladder Trauma, n = 57,886	Urethral Trauma, n = 38,065
**Males**	76,562 (70%)	14,397 (42%)	22,885 (40%)	35,910 (94%)
**Age (years)**	47 (24–65)	60 (48–72)	63 (47–76)	74 (58–83)
**Hospital stay (days)**	4 (2–10)	9 (3–18)	10 (6–17)	9 (4–18)
**Hypertension**	25,661 (23%)	12,721 (37%)	21,273 (37%)	18,870 (50%)
**Diabetes**	8817 (8.1%)	4092 (12%)	6808 (12%)	8091 (21%)
**Chronic kidney disease**	7770 (7.1%)	3186 (9.3%)	5535 (9.6%)	7725 (20%)
**Chronic heart failure**	4111 (3.8%)	1903 (5.5%)	4165 (7.2%)	8265 (22%)
**Acute kidney failure**	5496 (5%)	3065 (8.9%)	5277 (9.1%)	4507 (12%)
**Transfusion**	16,748 (15%)	7530 (22%)	14,358 (25%)	7006 (18%)
**ICU admission**	14,171 (13%)	4604 (13%)	8825 (15%)	4606 (12%)
**Mortality**	2618 (2.4%)	1106 (3.2%)	2988 (5.2%)	2454 (6.4%)
**Age group**				
<20	19,446 (18%)	349 (1%)	1244 (2.1%)	1500 (3.9%)
20–29	13,892 (13%)	806 (2.3%)	1777 (3.1%)	1180 (3.1%)
30–39	10,746 (9.8%)	2412 (7%)	5448 (9.4%)	1467 (3.9%)
40–49	13,124 (12%)	5431 (16%)	7542 (13%)	2194 (5.8%)
50–59	15,490 (14%)	6958 (20%)	8625 (15%)	3342 (8.8%)
60–69	13,605 (12%)	7257 (21%)	9978 (17%)	4792 (13%)
70–79	12,938 (12%)	7085 (21%)	12,470 (22%)	8911 (23%)
>80	10,124 (9.3%)	4031 (12%)	10,796 (19%)	14,660 (39%)

**Table 2:** Incidence of urinary tract trauma in Germany per 100,000 inhabitants.

Characteristic	Kidney Trauma	Ureteral Trauma	Urinary Bladder Trauma	Urethral Trauma
2005	8.49	1.32	2.48	1.62
2006	8.42	1.34	2.29	1.53
2007	7.91	1.41	2.33	1.63
2008	7.64	1.57	2.39	1.73
2009	7.77	1.57	2.51	1.98
2010	7.59	1.67	2.60	2.03
2011	7.78	1.90	2.86	2.08
2012	7.52	2.02	3.11	2.13
2013	7.35	2.18	3.38	2.22
2014	7.39	2.09	3.69	2.44
2015	7.27	2.31	3.95	2.58
2016	7.25	2.54	4.36	2.63
2017	7.16	2.59	4.71	2.73
2018	6.92	2.80	4.64	3.08
2019	6.21	3.05	4.93	3.08
2020	5.47	2.77	4.86	3.01
2021	5.12	2.68	4.88	3.14
2022	4.89	2.86	4.81	3.12
2023	4.91	2.94	5.37	3.37

## Data Availability

The original contributions presented in the study are included in the article, further inquiries can be directed to the corresponding authors.
